# The impact of temperature on the reproductive development, body condition and mortality of autumn migrating monarch butterflies in the laboratory

**DOI:** 10.1098/rsos.250343

**Published:** 2025-08-13

**Authors:** Michael Rich, Jasmine H. Kesselring, Amy Garcia, Danielle Wallin, Kenneth Michael Fedorka

**Affiliations:** ^1^ Department of Biology, University of Central Florida, Orlando, FL, USA

**Keywords:** migration, overwintering, *Ophryocystis elektroschirrha*, mature oocytes, reproductive diapause, climate change

## Abstract

Overwintering populations of the monarch butterfly, *Danaus plexippus*, have been in decline for the past 30 years. Several hypotheses for the decline have been proposed, including summer and winter habitat loss and migration mortality due to non-senescing milkweeds and the parasite *Ophryocystis elektroscirrha* (OE). However, the impact of climate change on migrant physiology, has been understudied. This is surprising because warmer temperatures will probably destabilize reproductive diapause, a physiological strategy central to migration and overwintering success. Here, we exposed wild-caught migrants to different field-realistic migratory temperatures under laboratory conditions for 30 days, followed by different overwintering temperatures until death. During the migratory phase, warmer temperatures reduced male body condition, increased male mortality, increased mating frequency and caused females to prematurely abandon their reproductive diapause/dormancy and invest in oocyte production in the absence of milkweed. Monarchs that experienced warm migratory conditions prior to overwintering also exhibited greater overwintering reproductive development and mortality. Overall, reproductive development and OE burden were the best predictors of death. These data suggest warm migratory temperatures significantly alter monarch physiology and fitness and provide a mechanism by which climate change could facilitate migratory failure, winter-breeding and overwintering mortality, all of which can decrease overwintering population size.

## 1. Introduction

One of the most well-known and well-documented animal migrations on the planet is undertaken by the monarch butterfly, *Danaus plexippus* [1]. In North America, *D. plexippus* is split by the Rocky Mountains into geographically distinct (but not genetically distinct due to gene flow [2,3]) eastern and western groups that breed between 30° N and 50° N latitude during spring and summer. Every year in late summer, a new adult generation emerges in reproductive diapause and migrates throughout September and October to overwintering sites in the mountains of central Mexico and the coastline of central/southern California [4,5]. They remain non-reproductive at these sites until early the following year when they initiate breeding and remigration northwards [6]. Both overwintering populations have exhibited declines over the past several decades [7–9], with a near 70% decline in the Mexican overwintering populations from 1993 to 2024 (based on the proportional difference in decadal averages of forest hectares occupied by overwintering monarchs between 1993–2002 and 2015–2024). This suggests North America is at risk of losing the migratory-overwintering monarch phenotype [10].

An abundance of research has sought insight into the initial and continued reasons for the overwintering decline, but significant gaps remain in our understanding of the phenomenon. The most well-investigated hypotheses include habitat loss in the summer and winter habitats and migratory failure in the autumn. Summer habitat loss is driven by urbanization, habitat fragmentation and an increased use of the herbicide glyphosate on agricultural crops (aka Roundup™), all of which have greatly reduced milkweed abundance [11–13]. However, summer-breeding populations appear relatively stable [14–16]. While stable breeding populations make the overwintering declines difficult to interpret, it does suggest that the continued declines are more likely the result of overwintering and/or migration failure. Logging in overwintering sites has clearly reduced forest area and density [17], exposing monarchs to a greater frequency of temperature extremes that can decrease survival [7,9,18]. But suitable habitat in Mexico’s Monarch Butterfly Biosphere Reserve remains unoccupied in lean overwintering years (e.g. 2012–2014), suggesting migration failure may be the most closely linked to monarch overwintering decline.

According to recent research, migratory populations have deteriorated over the past 17 years, as evidenced by reduced roost size along the migration route [19]. The main reason underlying this deterioration is currently unknown, though non-senescing (e.g. tropical) milkweeds have been suggested as a contributing factor. Such milkweeds may delay the onset of migration past its optimal timeframe or lure migrants to abandon migration and attempt reproduction, ultimately exposing them to freezing temperatures and mortality [20,21]. The protozoan parasite *Ophryocystis elektroschirrha* (hereafter referred to as OE) can also accumulate to higher levels on non-senescing milkweeds, which can weaken migrants by reducing flight competency [22] and increasing mortality [5,23]. Given that tropical milkweeds do not senesce and thereby accumulate a greater abundance of OE spores, many organizations have recommended against the sale of these plants.

Perhaps the most significant threat to monarchs is climate change [24,25]. Temperature has a large impact on insect physiology, behaviour, morphology, life history strategies and fitness [26,27]. Therefore, we should expect a changing climate to have a considerable impact on the monarch’s life cycle. Elevated summer temperatures under controlled laboratory conditions have been shown to improve monarch larval and milkweed growth rates [28], suggesting that warming temperatures may improve monarch abundance. However, the impact of higher temperatures on summer abundance depended on when and where during the breeding season these temperatures were experienced [29,30]. Moreover, niche modelling suggested monarch abundance may decline as breeding ranges expand northwards over the next 50 years [11,20,31]. Extreme high temperatures during larval development have also been shown to reduce starvation resistance and survival, while increasing wing deformities [32]. Niche modelling of overwintering sites indicated they may eventually become unsuitable due to a predicted increase in winter cool-weather precipitation [33] and a decoupling of suitable climatic conditions from dense, protected forest [34]. While numerous studies point to climate change impacting summer and winter populations, few have addressed how climate change will impact autumn migration ([35]; but see [11]).

The biological response to future autumn conditions has been an understudied aspect of climate change research [36]. Regarding the monarch butterfly, this is surprising as warming autumn temperatures may destabilize the diapause phenotype [35,37]. Diapause induction and metabolic rate in insects can be heavily influenced by temperature [38]. Thus, pre-migratory and migratory monarchs in warmer environments may invest in fewer lipids, storage proteins or cryoprotectants (e.g. glycerol) needed for migration/overwintering due to muddled diapause signalling (e.g. warm days with declining photoperiod), higher metabolic demands [39] or poor lipid storage efficiency [40]. Moreover, higher temperatures may cause some autumn monarchs to begin migration in a less recalcitrant state of diapause [41], making them prone to abandon migration and attempt reproduction [42]. This supposition is not without merit, as monarch butterflies in Australia exhibit a less restrictive diapause phenotype termed oligopause [41,43], distinguished by an abrupt return to reproductive development shortly after exposure to favourable thermal conditions and without the need for a prolonged refractory period characteristic of deep diapause. A similar abrupt termination pattern was also recorded for North American monarchs exposed to favourable thermal conditions in the laboratory [44], and many migrating monarchs collected along the migratory route have shown evidence of reproductive development [45]. Thus, North American monarchs appear to be in a flexible state of reproductive dormancy, like oligopause rather than deep diapause.

In addition to migration, warmer temperatures can also affect reproductive dormancy during overwintering. Colder temperatures during overwintering suppress insect metabolic rates and allow limited energy stores to be conserved. Warmer overwintering conditions could cause many monarchs to prematurely utilize their stores prior to the spring re-emergence of native milkweeds, condemning them to failure [37]. This is especially true for those in poor condition. Given the importance of migratory success and the potential impact of increased temperature on reproductive dormancy, controlled laboratory studies are needed to effectively assess the role of field-realistic autumn and winter conditions on monarch physiology and fitness [35].

Here, we examined the influence of simulated autumn and winter temperatures and photoperiod on eastern monarch butterfly migrants. Specifically, we exposed autumn migrants collected in Oklahoma to warm and cold migratory temperatures followed by warm and cold overwintering temperatures under photoperiod-appropriate laboratory conditions. The temperatures utilized were drawn from the naturally occurring distribution of temperatures experienced by monarchs over the past 70 years (National Oceanic and Atmospheric Administration (NOAA) National Centers for Environmental Information). We then assessed how these treatments affected monarch body condition, reproductive development and mortality. These data allowed us to better understand the hazard climate change may pose to migrating and overwintering monarchs.

## 2. Methods

### 2.1. Monarch collection

From 2 to 4 October 2023, 499 monarchs (*n* = 215 females and *n* = 284 males) were collected from El Reno, Oklahoma (USDA permit no. 526-23-236-10241). At the end of each collection day, monarchs were placed in glassine envelopes on site, boxed alongside icepacks and shipped overnight to the University of Central Florida. Immediately upon receipt, monarchs were placed in Percival incubators (model I-36 VL) 17.7|12.2°C (high|low, and a 11.35|12.25 day|night cycle) with ad libitum access to water and butterfly nectar (Birds Choice, universal product code (UPC): 789453300119). From 3 to 5 October 2023, monarchs were given a unique ID labelled on both ventral hindwings with a fine-tipped permanent marker, weighed and assessed for OE spores by pressing a 2 cm^2^ piece of clear tape to the ventral abdomen. The tape was then placed onto a microscope slide, labelled and set aside for future processing. On 6 October, after all monarchs were completely processed, they were randomly assigned to a ‘warm’ or ‘cold’ migratory treatment.

### 2.2. Migratory treatment

**Figure 1.**
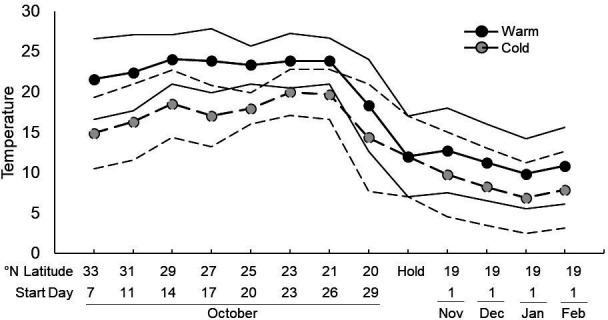
Migration-phase temperature regime. The thick solid line represents the average temperature of warm treatment, while the thin solid lines represent *T*_max_ and *T*_min_. The thick dashed line represents the average temperature of the cold treatment, while the thin dashed lines represent *T*_max_ and *T*_min_. See electronic supplementary material, table S1, for more details on locations.

To simulate migratory conditions, we projected where the source-migrant population would be over the subsequent four weeks after collection, as they travelled from the collection site in Oklahoma (on 4 October) to their final overwintering destination in Michoacán, Mexico (expected to arrive 1 November). Projections were derived from Journey North’s publicly available database that has tracked migratory movement since 1998 (https://maps.journeynorth.org/map/?map=monarch-peak-migration). Accordingly, we selected eight sites along the migratory route (electronic supplementary material, table S1). For each site, we obtained minimum and maximum daily temperatures (*T*_min_ and *T*_max_, respectively) from 1950 to 2022 for the dates the monarchs were expected to be at those locations. Temperature data were obtained from the NOAA National Centers for Environmental Information (https://www.ncei.noaa.gov/cdo-web/). Not all sites had complete temperature records for those years (average = 61 years; minimum = 44 years). For each site and date range, we calculated the first and third quartiles for both *T*_min_ and *T*_max_. Those temperatures formed the basis for our cold (*T*_min_ *Q*_1_ and *T*_max_ *Q*_1_) and warm (*T*_min_ *Q*_3_ and *T*_max_ *Q*_3_) migratory treatments and represent reasonable cold and warm migratory seasons expected under normal conditions. Considering that monarchs generally fly between 300 and 1200 m above sea level (higher altitude reduces mean total power expended for flight [46]), and temperatures decline by approximately 1°C for every 100 m rise in elevation [47], we subtracted 5°C from each *T*_max_ to approximate in-flight temperatures. Furthermore, migrants tend to seek out overnight roosts buffered from thermal extremes (P Cherubini 2023, personal communication). We therefore added 1°C to each *T*_min_ to obtain our final treatment temperatures (figure 1; electronic supplementary material, table S1).

From the adjusted *T*_min_ and *T*_max_ temperatures, we generated curves to approximate the sigmoidal pattern of daily temperature change. These curves were then programmed in 24-step increments into six Percival incubators (model I-36VL); three for each treatment. The light schedule was also programmed to simulate the expected daily photoperiod for each site and date (http://timeanddate.com) and used four natural spectrum fluorescent bulbs. New migratory location temperatures were programmed approximately every three days, and photoperiod was adjusted daily (electronic supplementary material, table S1). All collected monarchs were randomly assigned to one of four nylon mesh cages within each incubator (*n* = 24 cages; *n* = 20.8 monarchs of mixed sex per cage, *n* = 499 total monarchs). Cages were 60 × 40 × 40 cm (0.1 m^3^), containing two small petri dishes (5.5 cm diameter) with small cotton rounds (Walgreens Premium Cotton Rounds, UPC: 04 902 248 787), with one soaked in water and the other in butterfly nectar (Birds Choice, UPC: 789 453 300 119). Cages were rotated as a group mid-day between their treatment incubators, and water and nectar were completely replaced every three days. Work in our laboratory suggests that changing nectar and water every three days, as opposed to every day, does not impact mortality (KM Fedorka 2024, unpublished data).

One incubator in each treatment was designated for energetic ‘activity’. Within the activity incubators, each cage was outfitted at one end with two computer fans mounted within a wooden frame and covered in wire mesh to avoid contact with butterflies (electronic supplementary material, figure S1). Each fan was 12 × 12 × 2.4 cm and connected to a 12 V controller set to produce a 50 cubic feet per minute (CFM) horizontal airflow along the length of the cage (Easy Cloud EC12038 + Controller-1P). Fans were run for 2 h in the morning (10.00–12.00) before rotating cages, and 2 h in the afternoon (13.00–15.00) after rotating cages. Thus, each set of cages experienced the fans two out of every three days during the migratory phase. Each activity incubator used four natural spectrum fluorescent bulbs plus an extra bank of four fluorescent grow lights on one side. Fans were placed on the highlight side, causing monarchs to continually move towards the lights and fans (monarchs are positively phototactic). This caused monarchs to be more active by moving around the cage and wing flapping relative to their inactive counterparts in other incubators (activity was probably far less than experienced during migration). Activity incubators were not used to simulate migratory conditions or as a proxy for flight energetic expenditure. They were used simply to increase activity, as energetic activity such as flight has been shown to increase juvenile hormone inactivation [48], which would help maintain the reproductive diapause/dormancy phenotype.

Cages were monitored every day for death. As monarchs died throughout the migratory phase, they were weighed and stored at −20°C. At the end of the migratory phase (1 November 2023), all surviving monarchs were held at the same temperature (17°C|7°C high|low) and photoperiod (11.29|12.31 light|dark) for 24 h while they were re-weighed. The next day, surviving monarchs were randomly assigned to an overwintering treatment.

### 2.3. Overwintering treatment

The overwintering site for eastern monarchs is mostly in the Monarch Butterfly Biosphere Reserve, a dense mountain forest between 3000 and 3650 m elevation and approximately 120 km west of Mexico City, Mexico. Robust temperature data for this site were not available from NOAA. Instead, we used published records to estimate the *T*_min_ and *T*_max_ experienced by the monarchs from November to February [49,50]. From these averages, we subtracted 1°C to create our cold winter treatment and added 2°C to create our warm winter treatment (figure 1; electronic supplementary material, table S1). As before, we generated daily sigmoidal curves from the monthly estimates, programmed in 24-step increments. The light schedule was also programmed to simulate the expected photoperiod at that site and was modified daily (https://timeanddate.com). Incubators used only two natural spectrum bulbs to reduce light levels. In this phase of the experiment, each treatment was assigned two incubators, with two cages per incubator, each holding approximately 40 monarchs. Monarchs were held at higher densities to better approximate the cluster densities at overwintering sites. Each cage held two small petri dishes with cotton-soaked water and cotton-soaked butterfly nectar. Cages were rotated as a group mid-month among their treatment incubators, and water and nectar were replenished every seven days. Cages were monitored every day for death. As monarchs died, they were weighed and stored at −20°C. After approximately three months, the few surviving monarchs (*n* = 11) were censored (i.e. listed as ‘alive’ for survival analyses purposes), weighed and frozen.

### 2.4. Trait assessment

To assess reproductive development, female abdomens were dissected post-mortem and the number of ridged mature oocytes (MO) counted. In addition, mating males and females were recorded during the migratory and overwintering phases when cages were checked daily for mortality. Lifespan was estimated as the number of days alive since the start of the experiment. Parasite load was estimated by counting all OE spores present on the 2 cm^2^ tape. Considering that spores can be transferred between adults during roosting, we categorized individuals with fewer than 100 spores as contaminated [51] and set their spore count to zero. Initial body condition was assessed by dividing individual body weight at the start of the experiment by wing vein length. Wing vein size was estimated using ImageJ [52] as the distance between the CuA2 and M3 cells along the central wing vein of the left forewing. The length of this wing vein is positively correlated with total wing length (*r* = 0.77, *p* = 0.0014, *n* = 13), is not compromised by wing tip damage and is more repeatable than total wing length measured using a hand-held ruler. To this end, a digital image of each monarch was obtained during the migratory phase by briefly exposing them to CO_2_ and placing their dorsum on the glass of a digital scanner (Epson model B11B267201). To fully immobilize the butterfly and flatten the wing, microscope slides were placed on the wing’s ventral side. After a scanned image was obtained (1200 dpi), butterflies were returned to their cages. To assess change in condition, monarchs were weighed at the end of the migratory phase or when they died. Change in condition was calculated as a per cent change from initial condition (−1 × (1-current condition/initial condition)).

### 2.5. Statistical analysis

*Migratory phase.* To assess the impact of the migratory treatment on mortality, we utilized Cox proportional hazards mixed models (coxme R-package; [53]), as this was ‘time-to-event’ data. Those alive at the end of the migratory phase were censored and assigned a lifespan of 31 days (all monarchs were assigned an experimental start date of 2 October, and the migration phase ended 31 days later on 1 November). Possible model fixed variables included migratory treatment, sex, sex × migratory treatment, initial condition and OE count. Migratory incubator group (set of cages moved daily among the activity and non-activity incubators) and migratory cage were included as possible random variables. To assess models, we first determined the optimal random structure for a full fixed-effects model. Once the optimal random structure was determined, all possible fixed-effect models were then compared using the same random structure [54]. The model with the lowestAkaike information criterion corrected (AICc) score was selected.

To assess the impact of migratory treatment on condition change, we utilized linear mixed models (lme4 R-package; [55]) with a normal distribution (logit link). The same variables and model selection process as above were used, including an additional fixed variable ‘status’ to indicate if a monarch was alive or dead at the time of condition assessment. To assess the impact of migratory treatment on reproductive development, we used zero-inflated negative binomial distribution mixed models (glmmTMB R-package; [56]), as most females did not produce MO. Again, the same model variables and selection process were used, excluding the fixed variable sex and its interactions.

*Overwintering phase.* To assess the impact of overwintering treatment on mortality, condition and reproduction, we used the same analyses and AICc selection process as above. Possible model fixed variables included migratory treatment, overwintering treatment, sex, sex × migratory treatment, sex × overwintering treatment, migratory treatment × overwintering treatment, initial condition and OE count. Migratory cage group, overwintering cage group, migratory cage and overwinter cage were included as potential random effects. Once the appropriate model random structure was identified [54], the model with the lowest AICc score was selected. Note that 11 individuals were still alive at the end of the experiment and were censored for the mortality analysis.

For all migratory and overwintering analyses, model AICc scores and sample sizes appear in electronic supplementary material, table S2A–H. During model comparison, all similar models (ΔAICc < 2) were considered. In only one instance did a similar model produce slightly different results by adding significant terms (table 1A versus B). The alternative model is discussed. All analysis was conducted using R 4.4.1.

**Table 1.** The effect of reproductive investment (mature oocytes) and OE on female mortality during overwintering. ‘Trt’ = treatment, and ‘HR’ = hazard ratio.

source	HR	95% CI	*z*	*p*
A. all overwintering females—best AICc model (ΔAICc = 0.0)				
winter Trt (warm)	1.11	[0.68, 1.54]	0.49	0.622
initial condition	0.33	[−13, 14]	−0.2	0.878
mature oocytes	1.01	[1.007, 1.018]	4.5	<0.0001
B. all overwintering females—second best AICc model (ΔAICc = 0.9)				
winter Trt (warm)	1.12	[0.7, 1.6]	0.51	0.6101
initial condition	0.10	[−13, 14]	−0.3	0.7438
mature oocytes	1.01	[1.007, 1.018]	4.77	<0.0001
OE	1.00	[1.000, 1.002]	2.21	0.0272
mature oocytes × OE	1.00	[0.999, 0.000]	−2.3	0.0230
C. overwintering females without MOs				
OE	1.001	[1.0001, 1.0012]	2.3	0.0216
initial condition	0.62	[−18, 19]	−0.1	0.9608

## 3. Results

In total, 215 females and 284 males were used in the experiment. Upon collection from the field, males exhibited a larger average wing vein length than females (means + s.e.: 6.88 + 0.03 mm versus 6.75 + 0.03 mm, *p* = 0.0012), a greater weight (0.60 + 0.006 g versus 0.56 + 0.008 g, *p* = 0.0005) and better initial condition (0.088 + 0.001 g mm^−1^ versus 0.084 + 0.001 g mm^−1^, *p* < 0.0141). For both males and females, initial weight was positively correlated with wing vein length (Spearman *ρ* = 0.19 versus 0.21, respectively; all *p* < 0.05) and initial condition (Spearman *ρ* = 0.92 versus 0.93, respectively; all *p* < 0.0001). Males also exhibited a negative relationship between initial condition and wing vein length (Spearman *ρ* = −0.18, *p* = 0.0026).

**Figure 2.**
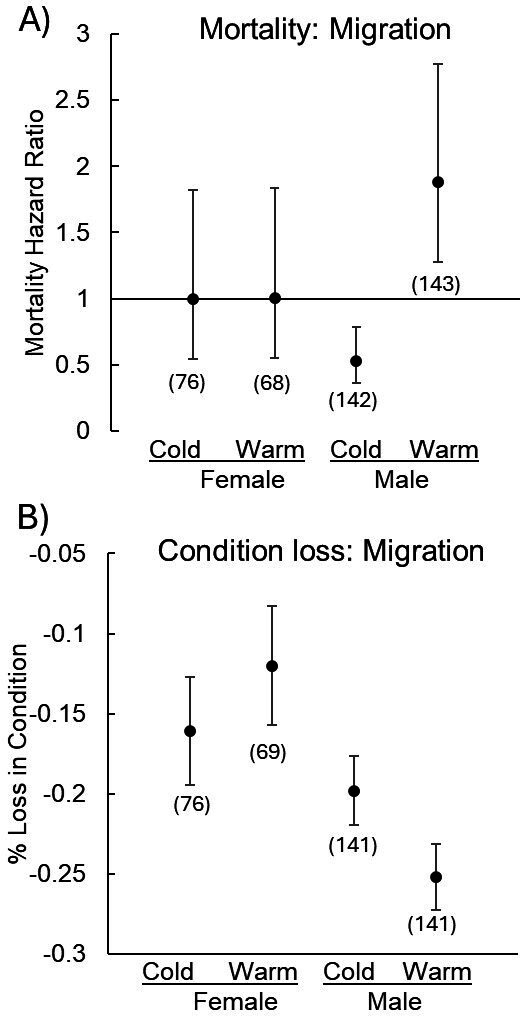
Effect of migration temperature on mortality risk and change in condition. (A) Males from the warm migratory treatment exhibited a greater mortality risk than did males from the cold treatment. No effect of treatment was detected for females. A hazard ratio of 1 indicates no difference between treatments in the risk of dying, while a hazard ratio greater than 1 indicates a greater risk of dying. (B) Warm treatment males exhibited a greater drop in condition than cold treatment. Females exhibited no difference. Sample sizes appear in parentheses. Estimates are LSmeans + 95% CI.

*Migratory phase.* The best AICc mortality model included a marginally significant sex-by-treatment interaction (*p* = 0.0818; electronic supplementary material, table S2A), prompting a separate analysis of the sexes. The best sex-specific models indicated that the warm migratory treatment significantly increased male mortality risk by 88% (hazard ratio + s.e. = 1.88 + 0.20) but did not impact female risk (table 2, figure 2A; see electronic supplementary material, figure S2 for survival curves). The best AICc model assessing condition loss also contained a significant sex-by-treatment interaction (*p* = 0.0013; electronic supplementary material, table S2B). The best sex-specific models showed that warm treatment males exhibited a greater decline in condition compared with cold treatment males, while females exhibited no difference (table 2, figure 2B). Furthermore, dead males and females exhibited a greater loss in condition than their counterparts that survived the migration period (male dead versus alive condition loss (means + s.e.: −0.25 + 0.01 versus −0.20 + 0.01; female dead versus alive condition loss: −0.17 + 0.02 versus −0.11 + 0.01). In both sexes, those in the highest initial condition exhibited the greatest drop in condition during migration (table 2).

**Table 2.** The effect of migratory treatment on mortality, condition and reproductive investment. Models are those with the lowest AICc score. ‘Trt’, treatment; ‘HR’, hazard ratio; ‘est’, regression estimate. Count and ZINB are the oocyte count (positive integers) and binomial count (oocytes present or absent) components of the zero-inflated negative binomial (ZINB) model, respectively.

mortality					
	source	HR	95% CI	*z*	*p*
♀	migratory Trt (warm)	1	[0.55, 1.84]	0.0	0.988
	initial condition	3807	[0, 7e+11]	0.8	0.396
♂	migratory Trt (warm)	1.88	[1.27, 2.77]	3.2	0.0015

condition					
	source	est	95% CI	*t*	*p*
♀	intercept	0.4	[0.3, 0.6]	6.9	<0.0001
	migratory Trt (warm)	0.04	[−0.01, 0.09]	1.7	0.0977
	status (dead)	−0.1	[−0.1, −0.00]	−2.1	0.0396
	initial condition	−6.9	[−8.4, −5.4]	−9.1	<0.0001
♂	intercept	0.2	[0.1, 0.3]	4.3	<0.0001
	migratory Trt (warm)	−0.05	[−0.08, −0.02]	−3.6	0.0045
	status (dead)	−0.04	[−0.07, −0.01]	−2.8	0.0004
	initial condition	−4.4	[−5.4, −3.3]	−8.3	<0.0001

**reproductive investment**					
	source	est	95% CI	*z*	*p*
count	intercept	2.5	[−0.3, 5.3]	1.8	0.0765
	migratory Trt (warm)	0.6	[−0.3, 1.4]	1.4	0.1755
	initial condition	13	[−17, 44]	0.9	0.3917
ZINB	intercept	4.1	[−0.6,8.8]	1.7	0.0871
	migratory Trt (warm)	−1.8	[−3.3, −0.3]	−2.3	0.0214
	initial condition	−27	[−78, 25]	−1.0	0.3041

**Figure 3.**
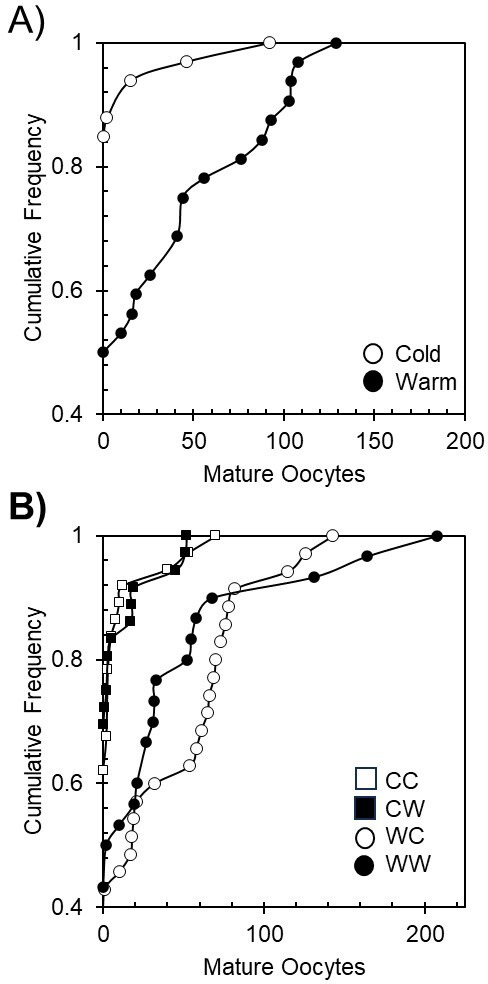
Reproductive investment during migration and overwintering. (A) The warm migratory treatment caused more females to produce oocytes and to a greater extent. (B) Females exposed to the warm migratory treatment followed by either a warm or cold overwintering treatment produced the most mature oocytes, while those first exposed to a cold migratory treatment followed by either a warm or cold overwintering treatment produced the least. CC, cold migratory, cold overwinter; CW, cold migratory, warm overwinter; WC, warm migratory, cold overwinter; WW, warm migratory, warm overwinter.

Migratory treatment also impacted reproductive development, with warm treatment females having a greater probability of producing MO than their cold treatment counterparts (table 2; figure 3A). Note that mature oocyte assessment is destructive and therefore only contains females that died during migration. In fact, 50% of warm treatment females that died produced MO compared with only 15% of cold females (*χ*^2^ = 11.5, *p* = 0.0007), resulting in dramatically different cumulative frequency distributions (figure 3A). The first female died on day 7 of the migration phase, and the first female with eggs died on day 10 (*n* = 44 MO), suggesting that exposure to warm migratory conditions resulted in an abrupt transition to reproduction. Males and females in the warm treatment were observed mating more often than in the cold treatment (for males: warm = 29 events, cold = 9 events; *χ*^2^ = 10.5, *p* = 0.0012). Despite the lack of milkweed, females in the warm treatment oviposited eggs on the cage substrate and those eggs were viable (*n* = approx. 150). No oviposition occurred in the cold treatment. The number and identity of females responsible for oviposition are unknown, but viable eggs were recovered in 4 of 12 warm treatment cages and 0 of 12 cold treatment cages. In short, warm migratory temperatures increased male mortality, male condition loss and male and female reproductive development.

**Figure 4.**
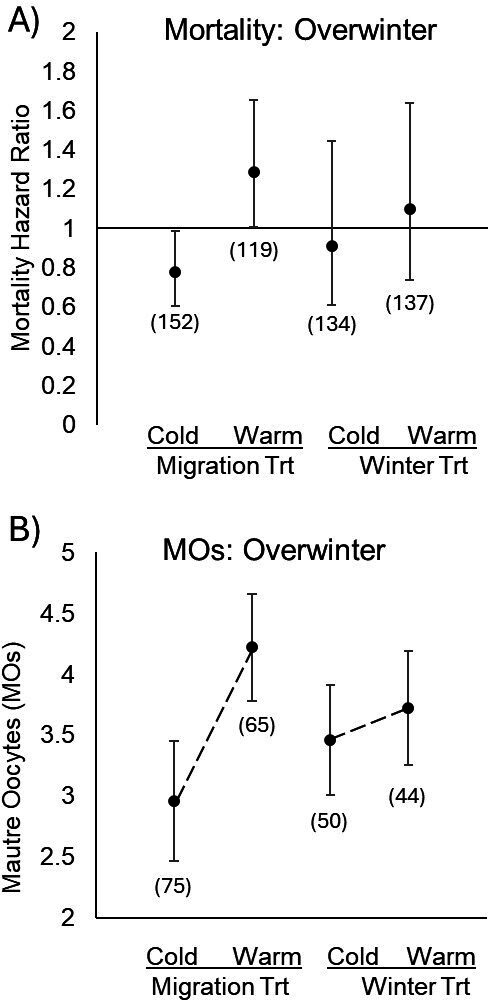
Effect of migration and overwintering treatments on mortality risk and reproductive investment. (A) Overwintering monarchs who first experienced a warm migratory treatment exhibited the greatest mortality risk, while overwintering treatment had no mortality effect and (B) Overwintering monarchs that first experienced the warm migratory treatment exhibited a robust increase in MO investment. Those in the warm overwintering treatment also exhibited a significant but smaller increase in MO investment. Estimates are LSmeans + s.e.

*Overwintering phase.* One hundred forty-nine females and 176 males (65%) survived the October migratory phase and were randomly assigned a warm or cold overwintering phase treatment. The lowest AICc mortality model indicated that the overwintering treatment did not impact monarch mortality (figure 4A, table 3). However, those in the overwintering phase that first experienced the warm migratory treatment exhibited, on average, a 28% greater risk of mortality (figure 4A, table 3; electronic supplementary material, figure S2), signifying that the temperature experienced during migration has a significant impact on overwintering survival. The lowest AICc condition model revealed that a change in condition was strongly negatively associated with initial condition for both sexes (table 3).

**Table 3.** The effect of overwintering and migratory treatment on mortality, condition and reproductive investment during overwintering. Models are those with the lowest AICc score. ‘Trt’, treatment; ‘HR’, hazard ratio; ‘est’, regression estimate. Count and ZINB are the oocyte count (positive integers) and binomial count (oocytes present or absent) components of the zero-inflated negative binomial (ZINB) model, respectively.

mortality		
random source	s.d.	distribution
Overwinter Inc Grp	0.16	Gaussian

source	HR	95% CI	*z*	*p*
migratory Trt (warm)	1.28	[1.03, 1.53]	1.95	0.0508
sex (male)	1.21	[0.95, 1.48]	1.44	0.1496
initial condition	0.44	[−7.9, 8.8]	−0.19	0.8475

condition					
	source	est	95% CI	*t*	*p*
♀	intercept	0.25837	[0.06, 0.46]	2.527	0.0135
	migratory Trt (warm)	0.06461	[−0.00, 0.13]	1.842	0.0692
	winter Trt (warm)	−0.05183	[−0.12, 0.02]	−1.49	0.1403
	initial condition	−6.17245	[−8.4, −3.9]	−5.32	<0.0001
♂	intercept	0.02059	[−0.12, 0.16]	0.285	0.776
	winter Trt (warm)	0.03661	[−0.01, 0.08]	1.574	0.117
	initial condition	−4.05577	[−5.6, −2.5]	−4.99	<0.0001

reproductive investment					
	source	est	95% CI	z	p
count	intercept	4.02	[1.9, 6.1]	3.7	0.0002
	migratory Trt (warm)	1.26	[.6, 1.9]	3.7	0.0002
	winter Trt (warm)	0.26	[−0.4, 0.9]	0.8	0.4393
	initial condition	−14.46	[−38, 9]	−1.2	0.2396
ZINB	intercept	1.17	[−1.2, 3.6]	0.9	0.3430
	migratory Trt (warm)	−0.83	[−1.7, 0.05]	−1.8	0.0663
	winter Trt (warm)	−0.25	[−1.1, 0.6]	−0.6	0.5770
	initial condition	−6.62	[−34, 21]	−0.5	0.6379

Regarding reproductive development, the overwintering temperature had no impact on the production of MO. However, the migratory temperature experienced by females prior to overwintering did impact oocyte production, with warmer migratory temperatures inducing females to initiate reproductive development earlier than cold migratory temperatures (table 3, figure 4B). Few matings were witnessed during the overwintering phase, and no difference in treatment was detected (warm = 4 events, cold = 0 events; *p* = 0.9952).

Given the associations between warm temperatures, mortality and reproductive development seen in both the migratory and overwintering phases, we asked if reproductive development could drive mortality in females. This question is motivated by the well-founded life history trade-off between reproduction and survival [57]. To assess this question, we readdressed our overwinter mortality analysis by including MO production as a potential independent variable and selected the best AICc model. We found that MO investment was the single best predictor of overwintering female mortality, with greater oocyte production leading to greater mortality risk (table 1A; electronic supplementary material, table S2G; note: this included all overwintering females). The next best AICc model (ΔAICc = 0.87) supported the above result. It also suggested that increased OE levels increased female mortality (table 1B). Regarding overwintering females that did not produce MO, OE burden was the only predictor of mortality (table 1C). When male mortality was re-examined without female data, no impact of the migratory treatment on overwintering mortality was detected. Overall, these patterns suggest that warm temperatures during migration initiated reproductive development in females. In turn, reproductive development and/or OE infection level increased female overwintering mortality risk.

## 4. Discussion

Monarch butterflies migrate extraordinary distances to avoid prolonged winter temperatures that threaten survival. While at their overwintering sites, they remain relatively inactive until favourable climatic conditions return in early spring. To meet these energetic demands, monarchs start migration in a state of reproductive diapause/dormancy to maximize resource allocation towards migration and overwinter survival. It is important to note that temperature plays an important role in the onset of diapause [4], diapause termination [41,43] and the rate at which limited resources are acquired and utilized during diapause [37]. With this in mind, we assessed the influence of temperature on monarch survival, condition and reproductive state during migration and overwintering. We found that warmer migratory temperatures increased male condition loss, male mortality risk and reproductive development in both males (increased mating rates) and females (increased MO incidence and mating rates). The impact of migratory temperature also extended into the overwintering phase, with those from the warm migratory treatment exhibiting the greatest mortality risk and reproductive development. Furthermore, reproductive development during overwintering and/or OE burden were the greatest predictors of mortality. Thus, our results suggest that warming temperatures in the field should have an impact on monarch migration and overwintering success.

To date, several mechanisms have been proposed to account for migration failure, including OE parasites impairing flight capacity and survival [5,22,23], reduced nectar availability [14,58] and tropical milkweeds promoting reproduction over migration [21]. Our data support the recent suggestion that warm temperatures can disrupt migration and lead to winter-breeding (i.e. reproduction that occurs past the migratory season [42,59,60]). Importantly, we found that reproductive development in migrants can be initiated quickly and in the absence of milkweed. Our data also contribute to our understanding of overwinter success. Females that experienced a warm migration exhibited greater reproductive development, which was a strong predictor of overwintering mortality. OE burden, which has generally been discussed as a mechanism for migration failure [5], was also a strong predictor of overwinter mortality, at least for females that did not reproductively invest. It may be that monarchs with higher OE burdens or early reproductive development induced by a warm migration depart their overwintering sites prematurely to attempt reproduction, the success of which would depend on the local winter climatic conditions and the phenology of local milkweed. If climates warm just enough to initiate reproduction, but not enough to initiate milkweed re-emergence, then these monarchs may become caught in an ecological trap that diminishes overwintering success, although other less catastrophic scenarios may occur.

Considering that temperatures are predicted to keep rising throughout North America, the migration-overwintering life stage may begin to transition to a truncated migration-winter breeding life stage [59,61]. A glimpse of this possible future was seen in the US western monarch population crash during the autumn of 2020 [8]. As California experienced record high migratory temperatures, a record low number of migrants arrived at the overwintering sites (less than 1% of the previous decade’s average abundance). Furthermore, a record high number of breeding adults, eggs and larvae were sighted near San Francisco that December, suggesting that some of the migrants switched to a winter-breeding strategy [60]. The following year, cooler migratory temperatures returned, as did a robust population of overwintering monarchs (estimated at over 240 000; [59]). The source of these monarchs is unclear, but could have been derived from the descendants of the winter-breeders [42,60], descendants of cryptic overwintering populations in California or remigrants from Mexico. If they are the descendants of winter-breeders, then transition to a truncated migration and winter-breeding life stage is viable. Since at least the mid-1960s, winter-breeding populations have arisen throughout the US Gulf Coast and southwestern US [62,63], which may be in part due to a warming climate [61]. An increase in winter breeders whose descendants expand northwards in spring is also consistent with the current observations of overwintering population decline [64] but stable summer-breeding populations [14,15]. As such, future research should focus on the fate of winter-breeding populations and how they may contribute to future northern migratory populations.

As stated, our data suggest warm migratory temperatures induce reproductive development independent of milkweed, which could cause monarchs to truncate migration and drop out even when milkweed is not present. If reproductively active dropouts (aka partial migrants) are to be successful, however, they must eventually find milkweed. Tropical milkweed that persists north of the overwinter site in Mexico could rescue dropouts from certain death. However, tropical milkweed has been shown to increase the transmission of OE spores, which reduces monarch condition and fitness [5,22]. This makes using tropical milkweed as a conservation tool controversial. That said, year-round monarch populations (e.g. Miami, Florida) appear to persist on tropical milkweed without extirpation [65]. It may be that the cost of using tropical milkweed to rescue dropout migrants (i.e. increased mortality rates) is overshadowed by the cost of truncated migration with no milkweed (i.e. absolute mortality). Although tropical milkweed has been suggested to entice migrants in reproductive diapause/dormancy to leave migration and reproduce [21], preliminary work in our laboratory suggests that the warm temperature experienced during migration without milkweed is at least as great a stimulus for reproductive development as milkweed exposure during migration (KM Fedorka, 2025, unpublished data). To be clear, we are not advocating planting tropical milkweed as a conservation effort. However, future work should assess the above scenario to determine if properly managed tropical milkweed could serve as a bridge between dwindling overwintering colonies and growing winter-breeding colonies resulting from a changing climate.

Although our experimental design provided robust results, extrapolation to natural monarch populations must be made with caution. Laboratory conditions cannot fully capture natural conditions. For instance, keeping monarchs caged during our experimental migration phase is unnatural and could possibly promote reproductive development by keeping monarchs inactive, which could allow juvenile hormone titres to increase [48]. We attempted to minimize this by running monarchs through our ‘activity’ incubators. Even though caged monarchs were a necessary aspect of the experimental design, most females (58%) remained non-reproductive upon their death. An alternative design might be annual collections during migration to correlate reproductive development and migration temperature in the field. However, this design also has limitations, as one might miss those invested in reproduction that have already left the migratory path in search of reproductive opportunities.

It should also be noted that the laboratory monarchs experienced a greater mortality rate than expected, with only 3.4% of the overwintering population surviving until 1 February 2024. The heightened mortality rate could have been due to at least three causes. First, we collected migrants midway through their migration in Oklahoma. Due to variation in body condition, not all migrants typically complete the migration journey [5]. Thus, much of the mortality in our experiment probably represents individuals destined to migratory failure. Second, the autumn 2023 migrating monarchs (from where our experimental population was derived) established the second smallest overwintering population on record in Mexico at just 0.9 hectares [66]. Such a small size suggests that the migrating monarchs may have been somehow different. They may have been in unusually poor condition or in a relatively shallow state of diapause, leading to the high mortality and/or high reproductive response to temperature. Third, we suspect our husbandry during the overwintering phase could have increased the potential for cold injury. In nature, overwintering monarchs are often positioned or move to gain some amount of dappled sunlight to regulate body temperature, something not available in a laboratory environment. This may have kept body temperature low enough to reduce osmoregulatory capacity, leading to injury [67]. Regardless, we show here that field-realistic temperatures expressed under photoperiod-appropriate laboratory conditions have a significant impact on the initiation of reproductive development, body condition and mortality risk. While this provides insight, how natural conditions impact natural populations must still be thoroughly investigated.

In summary, our results suggest that a warming climate will alter monarch physiology and fitness during migration and overwintering. Specifically, warmer temperatures will reduce condition and increase reproductive development and mortality. If early reproductive development alters reproductive behaviour, then monarchs may forsake migration or overwintering to reproduce. Our data are consistent with the supposition that the North American monarch may eventually transition to partial migration and winter-breeding as the climate continues to warm [8,61].

## Data Availability

Data are at Dryad [68]. Supplementary material is available online [69].
